# Morphologic and biometric evaluation of chick embryo eyes *in ovo* using 7 Tesla MRI

**DOI:** 10.1038/s41598-017-02755-4

**Published:** 2017-06-01

**Authors:** Tobias Lindner, Ronja Klose, Felix Streckenbach, Thomas Stahnke, Stefan Hadlich, Jens-Peter Kühn, Rudolf F. Guthoff, Andreas Wree, Anne-Marie Neumann, Marcus Frank, Änne Glass, Sönke Langner, Oliver Stachs

**Affiliations:** 1 Core Facility Multimodal Small Animal Imaging, Rostock University Medical Center, Rostock, Germany; 2 Department of Ophthalmology, Rostock University Medical Center, Rostock, Germany; 3 Institute for Diagnostic Radiology and Neuroradiology, University Medicine Greifswald, Greifswald, Germany; 4 Institute of Anatomy, Rostock University Medical Center, Rostock, Germany; 5 Medical Biology and Electron Microscopy Centre, Rostock University Medical Center, Rostock, Germany; 6 Institute for Biostatistics and Informatics in Medicine and Ageing Research, Rostock University Medical Center, Rostock, Germany; 7 Institut und Poliklinik für Radiologische Diagnostik, Carl Gustav Carus University, Dresden, Germany

## Abstract

The purposes of this study were (1) to characterize embryonic eye development during incubation *in ovo* and (2) to analyze the putative influence of repetitive ultrahigh-field MRI (UHF-MRI) measurements on this development. A population of 38 fertilized chicken eggs was divided into two sub-groups: two eggs (Group A) were examined repeatedly during the developmental period from embryonic day 1 (E1) to embryonic day 20 (E20) to evaluate the influence of daily MRI scanning. A second larger group of 36 eggs was examined pairwise on one day only, from E3 to E20, and the embryos were sacrificed immediately after MR imaging (Group B). Fast T2-weighted MR sequences provided biometric data on the eye with an in-plane resolution of 74 μm. The data show rapid growth of the eye with a steep increase in intraocular dimensions in all axis directions and in eyeball volume during initial development up to E10, followed by a phase of reduced growth rate in later developmental stages. Comparison of the two groups revealed no differences in ocular development.

## Introduction

The developing chick is an excellent and favored model for studies in the field of embryology research^1–3^. Bain *et al*. have observed that ‘all of the developing chick’s requirements, with the exception of oxygen and heat, are provided by the egg contents and the surrounding eggshell’^1^. Due to this fact and the ready accessibility and economical availability of fertilized chicken eggs, the *in ovo* embryo has become a widely used animal model in the basic and applied sciences^1, 3^, achieving particularly well-established status in the field of ophthalmological research^4–6^. For example, chick embryos have already been useful in research into the hypoxic cellular response^4^, the invagination of the optic vesicles^5^ and the effect of green LED light stimuli on post-hatch growth and eye development^6^.

According to the developmental staging series published by Hamburger and Hamilton^7^, chick ocular development starts at embryonic stage 9 (i.e., 29–33 hours after fertilization) with the formation of the optic vessels. The lens-placode is present at stage 14 (50–53 hours) and the optic cup is entirely shaped at stage 15 (50–55 hours). Embryos are usually sacrificed at different time points for histology, growth measurements and further examinations, a practice that precludes longitudinal assessments of the development of a specific embryo *in vivo* ^2, 4, 5, 8^. In some previous studies involving magnetic resonance imaging (MRI), avian embryos were fixed prior to imaging of different tissues and the eye in order to obtain good image quality and precise measurements with combined use of contrast agents^2, 8, 9^. MRI has also been commonly used for *in vivo* imaging of chick and quail embryos^1, 10, 11^, in principle permitting repeated observations of the same embryo^1^, and to assess the development of the chick eye. However, previous studies have been limited by their spatial in-plane resolution^12^, their invasiveness^2, 8, 13^ or their incomplete coverage of embryonic development^1, 2, 8^.

Ultrahigh-field MRI (UHF-MRI) with spatial resolution of the order of <100 μm is known as MR microscopy (MRM)^14, 15^. Because UHF-MRI provides anatomical images at high quality with excellent resolution comparable to conventional histology^16^, it is a logical step to apply this technique to the study of avian embryology. However, the drawback is that the scan time becomes quite long and, especially at later developmental stages, the embryo has to be cooled externally to reduce chick motion artifacts.

The purpose of the present study was to evaluate the feasibility of MR imaging with an in-plane resolution of <100 μm for continuous *in vivo* high-resolution assessment of the development of the chick eye *in ovo* and to examine the impact of repeated imaging and cooling on embryo development.

## Materials and Methods

### Chick embryo incubation

All animals were handled in accordance with the ARVO Statement for the Use of Animals in Ophthalmic and Vision Research and the experiments are compliant with national animal welfare legislation. Thirty-eight fertilized chicken eggs (White Leghorn) were obtained from a commercial hatchery (Valo BioMedia, Osterholz-Scharmbeck, Germany) and stored at room temperature (20 °C) for three days prior to the start of incubation, which was performed in an incubator with automated egg-turning (HEKA-Turbo 168, HEKA, Rietberg, Germany) at 37.8 °C and 60% relative humidity with 12 turns per day. All thirty-eight eggs were incubated simultaneously up to embryonic day 20 (E20). Eggs were scanned using MRI at 7.1 Tesla as follows: two eggs were scanned every day between E1 and E20 (Group A) and 36 eggs were scanned at only one time point (Group B) from E3 up to E20 (two eggs at each time point).

All MRI examinations were performed *in ovo* and none of the embryos hatched. Incubation was terminated at the indicated time points, the eggs were opened and third toe length was determined after embryo decapitation to control for correct embryonic development, according to the staging criteria of Hamburger and Hamilton^7^.

### MR imaging

**Figure 1:**
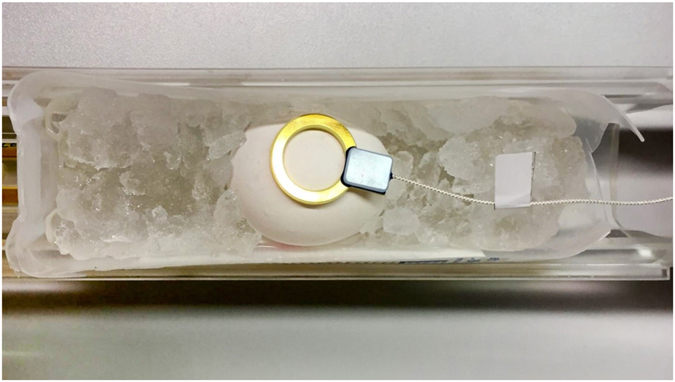
Egg with attached S1-torodial coil (golden ring) on a bed of crushed ice in a small plastic tray during cooling before imaging.

*In vivo* MR imaging was performed on a 7.1 T MRI scanner (ClinScan, Bruker Biospin, Ettlingen, Germany) with a bore size of 13 cm using a 16-channel volume coil (rat body coil) and a small surface loop coil of 30 mm diameter (s1 coil, Bruker Biospin) for signal detection (Fig. 1). During the first ten days, a fast T2-weighted (T2w) localizer was acquired using the 16-channel volume coil to identify chick position inside the egg. If necessary, the position of the egg was corrected and the actual position of the eyes of the embryo was marked on the outer surface of the egg for faster localization on follow-up imaging and actual data acquisition by the surface coil. Due to the increased size of the embryo from E11, fast T2w localizers were acquired with the surface coil and if necessary, the position of the coil was corrected. Slicing was adjusted with localizers until parallel and symmetrical visibility of left and right lenses was achieved. Afterward, high-resolution T2w turbo spin-echo (TSE) images of the orbits were acquired in two orthogonal planes. Imaging parameters were: TR 2100 ms, TE 48 ms, turbo factor 7, field of view (FoV) 38 × 38 mm with a slice thickness of 700 µm, and no partial-Fourier acceleration. With a matrix size of 512 × 512 interpolated with zero filling to 1024 × 1024, the in-plane resolution was 74 × 74 µm. The number of slices was adjusted to cover the entire orbit in the axial and coronal planes with a minimum number of 12 slices. Acquisition time depended on the number of slices and ranged between 12 minutes 15 seconds and 18 minutes 23 seconds.

For UHF-MRI, eggs older than E10 were bedded on crushed ice 10 minutes prior to the start and during MRI for 50–70 minutes in total to reduce embryo movement and associated motion artifacts (Fig. 1). It was deemed sufficient to start moderate cooling at E10 because no effects on image quality due to embryo movement were identified at earlier stages. MRI itself was then performed at room temperature (22.4 ± 1.2 °C) within the bore of the scanner.

### Measurement of embryonic third toe length

After MRI the eggs from Group B were carefully opened, the embryos were killed by decapitation, and third toe length was measured. Third toe length is defined as the distance between the metatarsal joint and the tip of claw^7^. Measurements were taken manually from E10 to E20 to determine the correct stage of development in comparison with reference values published in the literature^8,17^.

### Image analysis of chick eyes

**Figure 2:**
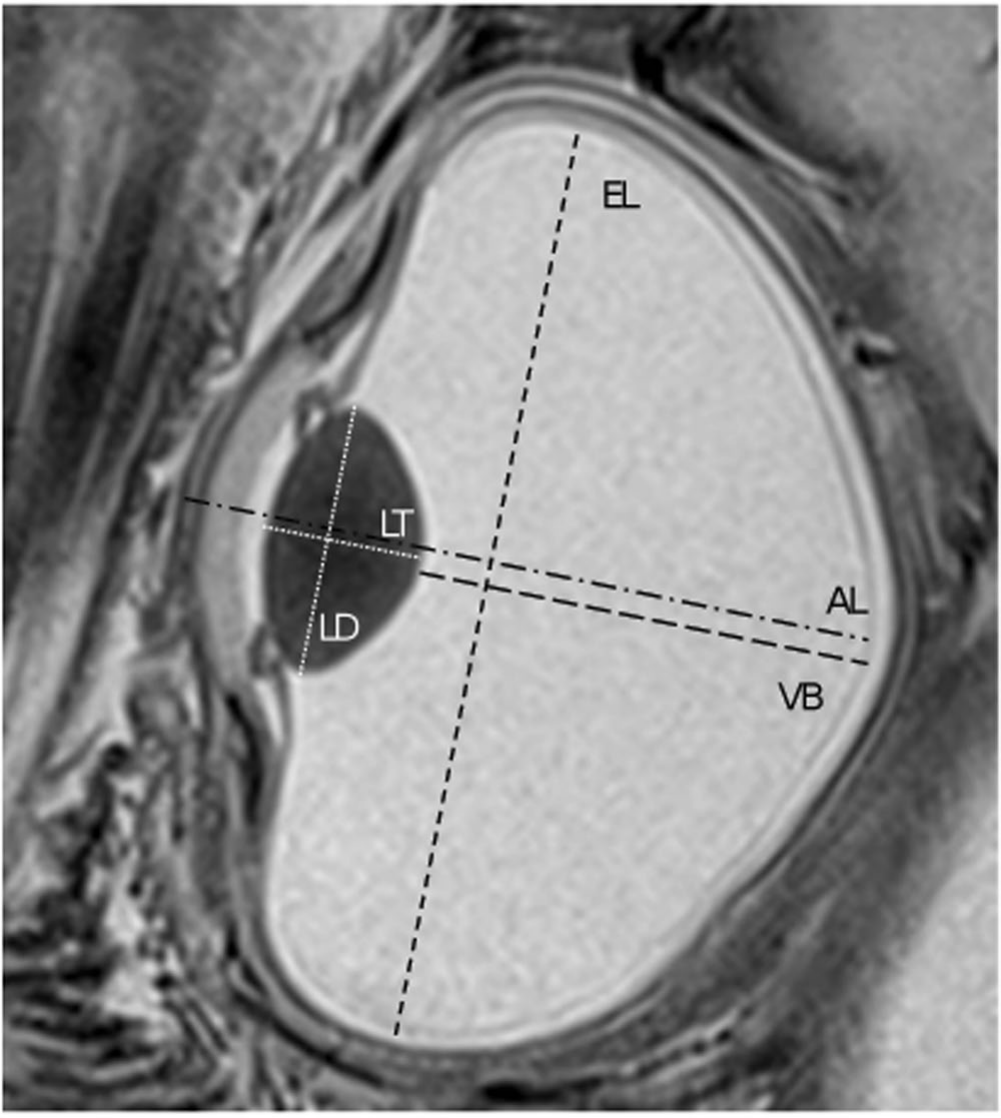
T2-weighted *in vivo* MR image of a chick embryo eye with intraocular dimensions depicted here schematically, as measured using OsiriX^©^: axial length of the eyeball (AL), equatorial length of the eyeball (EL), lens thickness (LT), lens diameter (LD) as well as vitreous body distance (VB).

For image evaluation and ocular biometry, MR datasets were transferred to OsiriX^©^ 7.5 (Pixmeo, Geneva, Switzerland). The following ocular measurements were performed (see Fig. 2): axial length (AL, posterior cornea to retina), equatorial length (EL, upper to lower pole of the eyeball including cornea), axial lens thickness (LT), lens diameter (LD), vitreous body distance (VB) and length of pecten oculi (PL). Mean values were calculated from left eye and right eye for each egg and hence we used only one value per egg for data analysis. The contour of the eyeball was defined manually by drawing a region of interest in every image where the eyeball was visible. The volume of the eyeball was then computed using the ROI volume calculation algorithm provided by OsiriX^©^. The circumferential surface of the eye therefore had to be taken from every individual slice.

### Tissue preparation and HE staining for ganglion cell counting

Eyeballs from Groups A and B (n = 2 in each case) were fixed at E20 in 4% PFA for 1 to 3 days and washed with PBS. Whole tissue was dehydrated in an ascending ethanol series, cleared with xylene, and infiltrated with liquid paraffin. Consecutive 5-µm thick sections were cut, and every 100^th^ section was stained with Mayer’s hematoxylin for 7–10 minutes, and cleaned with 1% hydrochloric acid in 70% ethanol for a few seconds. After washing with tap water for 15–30 minutes and brief rinsing in distilled water, sections were counterstained with 1% eosin for up to 3 minutes and rinsed again. Following dehydration in an ascending ethanol series and xylene clearance, the sections were covered with the mounting medium Entellan^®^ (Merck, Darmstadt, Germany) and coverslips. Cells of the retinal ganglion layer were counted under the microscope by evaluating all nuclei, and the total cell number of the retinal ganglion layer was estimated using Cavalieri’s method.

### Statistical analysis

All data were stored and analyzed using the SPSS statistical package 23.0 (SPSS Inc. Chicago, Illinois, USA). Descriptive statistics were computed for continuous variables, including mean and standard deviations. Because the Kolmogorov-Smirnov test did not reject the normal distribution hypothesis, testing for differences in continuous variables between study groups was accomplished with a 2-sample *t* test using the Bonferroni correction. For several parameters, evolution over time was described using specific linear or nonlinear regression analyses with best curve-fitting according to the coefficient of determination.

## Results

### General growth of chick embryo

**Figure 3:**
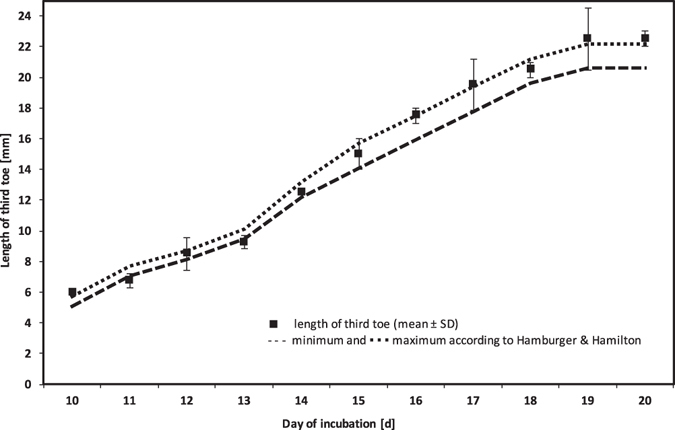
Length of third toe (mm) for chick embryos from Group B in relation to reference values established by Hamburger & Hamilton^7^.

All 38 fertilized chicken eggs were successfully incubated for analysis by MRI. Based on Group B, the length of the third toe was used as a reference mark for developmental staging from E10 (Hamburger and Hamilton (HH) stage 36) to E20 (HH stage 45). The results of our measurements in comparison with the reference values of Hamburger and Hamilton are illustrated in Fig. 3. The values (n = 2) collected from the examined embryos in Group B are for the most part located within the reference range.

**Figure 4:**
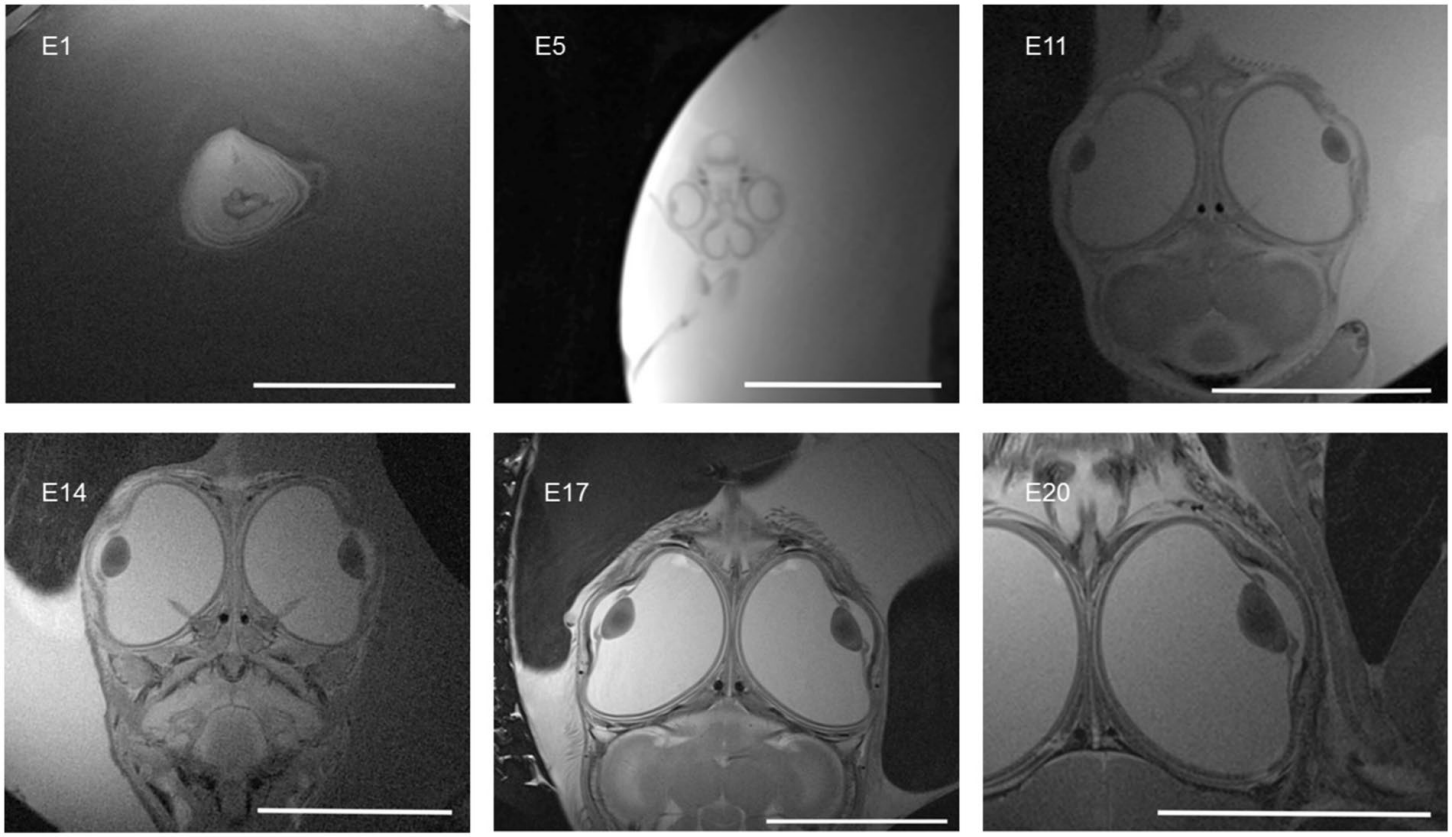
Examples of T2-weighted *in vivo* MR images (TE/TR: 48/2100 ms, FoV: 38 × 38 mm, slice thickness: 700 µm; matrix size: 512 × 512 interpolated to 1024 × 1024; in-plane resolution: 74 × 74 µm) of a chick embryo *in ovo* at different time points (E1–E20) during the development cycle. Each scale bar represents 10 mm.

A representative overview of the collected data on several days in the embryonic period is presented in Fig. 4 demonstrating that intraocular structures such as the lens, pecten oculi, ciliary body and the anterior and posterior chamber can be identified in T2w images. Embryonic structures were identified using MRI at E1, whereas ocular compartments were first identified at E4 (images not presented). Ocular biometric data can first be obtained at E5. The lens is the first intraocular anatomic structure to appear and it can be seen from E5. The ciliary body, which fixes the lens in the ciliary muscle, is visible from E10. The pecten oculi, a specific structure in the avian eye which contains vessels to supply the vitreous body, first appears at E8, but can be measured accurately from E12.

### Cooling and MRI influence

**Figure 5:**
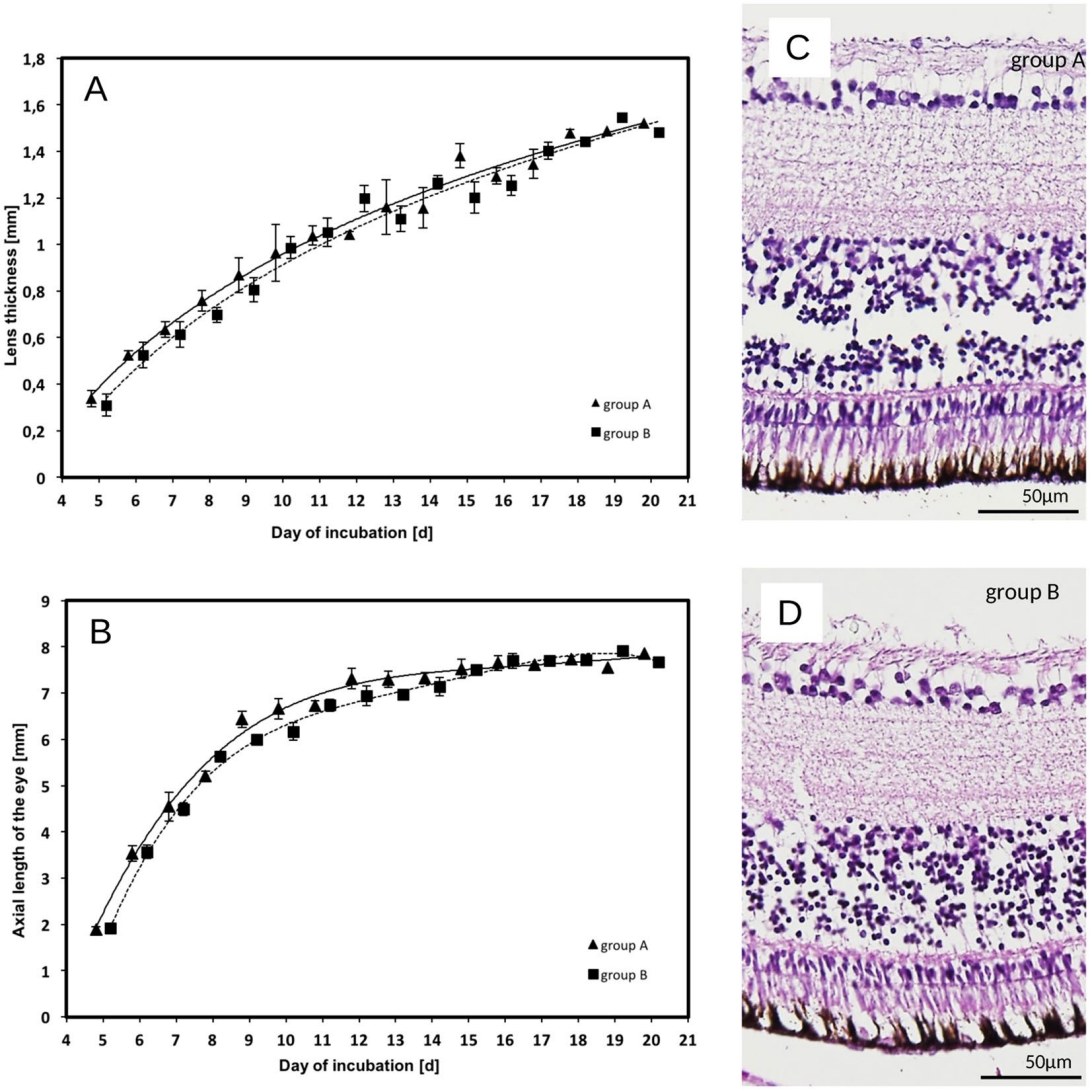
(**A** and **B**) Lens thickness and axial length of the eyeball in Groups A and B by day of incubation starting from E5 (note: data points ▲ and ■ are shifted slightly for each day to prevent marker overlap). (**C** and **D**) Examples of HE staining of the E20 retina for Group A and B for retinal ganglion cell counting.

To evaluate whether daily MRI scanning with cooling of the egg has any influence on embryonic growth, and specifically on the development of the eye, two intraocular dimensions (axial length of the eyeball (AL) and lens thickness (LT)) were compared between Groups A and B (Fig. 5). Intraocular dimensions were quantified in Groups A and B from E5 onward. In reality, closely corresponding values were obtained for both groups, with a maximum difference in mean axial length values observed at E10 (0.49 mm) but becoming minimal toward the end of incubation.

Curve-fitting using a nonlinear regression model yielded an excellent fit for lens thickness

1 $$LT=a+b\times \,\mathrm{ln}(day\,of\,incubation+1).$$

In detail, the equations were:

$$L{T}_{A}=-1.289+0.927\times \,\mathrm{ln}(day\,of\,incubation+1),({{\rm{r}}}^{2}=0.987)$$

and

$$L{T}_{B}=-\,1.339+0.944\times \,{\rm{l}}{\rm{n}}(dayof\,incubation+1),({{\rm{r}}}^{2}=0.977)$$

with very similar values for the parameters *a* and *b*. At each time point the 2-sample *t* test (with Bonferroni correction) clearly showed no significant differences between Groups A and B.

For axial length of the eyeball an excellent fit was obtained with the cubic model

2 $$AL=a+b\times day\,of\,incubation+c\times day\,of\,incubatio{n}^{2}+d\times day\,of\,incubatio{n}^{3}.$$

The respective equations for Groups A and B were:  $A{L}_{A}=-9.47+3.20\times day\,of\,incubation$ $-\,0.2014\times day\,of\,incubatio{n}^{2}\,+\,0.004241\times day\,of\,incubatio{n}^{3},({{\rm{r}}}^{2}=0.992)$ and

$$\begin{array}{ccc}A{L}_{B} & = & -7.77+2.75\times day\,of\,incubation-0.1681\times day\,of\,incubatio{n}^{2}\\  &  & +\,0.003498\times day\,of\,incubatio{n}^{3},({{\rm{r}}}^{2}=0.968).\end{array}$$

For all time points the 2-sample *t* test did not reject the equality hypothesis.

Furthermore, total cell counts (mean of two eyes from different eggs) were estimated in the chick embryo retinal ganglion layer at E20 in both experimental conditions, yielding 3.79 × 10^6^ ± 0.127 × 10^6^ in Group A and 3.85 × 10^6^ ± 0.200 × 10^6^ in Group B (Fig. 5) (2-sample *t* test: p = 0.754).

### Monitoring ocular growth

**Figure 6:**
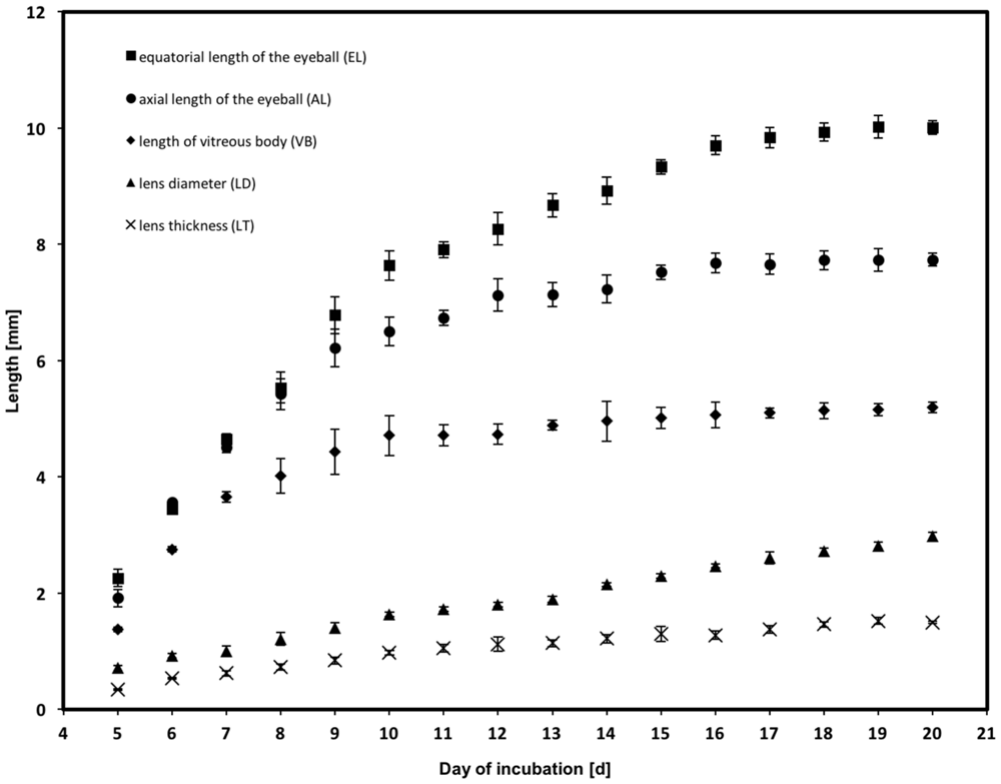
Changes in biometric dimensions EL, AL, VB, LD and LT over the incubation period, as measured by MRI.

As mentioned above, intraocular structures were first observed at E4 and during all subsequent stages. All measurements were acquired separately for each day of incubation on both eyes of each chick embryo, illustrating the different growth phases of the underlying structures (Fig. 6). Approximately linear growth was noted for lens diameter and lens thickness during development from E5 to E20 with

3 $$lens\,diameter=0.1504\times day\,of\,incubation+0.015221,({{\rm{r}}}^{2}=0.995),$$

and

4 $$lens\,thickness=0.0741\times day\,of\,incubation+0.1339,({{\rm{r}}}^{2}=0.957),$$

while vitreous body length, axial length of the eyeball and equatorial length of the eyeball displayed higher growth rates until E10 followed by moderate growth thereafter. Best curve fitting was achieved with the following cubic models:

5 $$\begin{array}{ccc}vitreous\,body\,length & = & -6.90+2.45\times day\,of\,incubation\\  &  & -\,0.1667\times day\,of\,incubatio{n}^{2}+0.003739\\  &  & \times \,day\,of\,incubatio{n}^{3},({{\rm{r}}}^{2}=0.978),\end{array}$$

6 $$\begin{array}{ccc}axial\,length\,\,of\,the\,eyeball & = & -8.65+2.98\times day\,of\,incubation-0.1852\\  &  & \times \,day\,of\,incubatio{n}^{2}+0.003872\\  &  & \times \,day\,of\,incubatio{n}^{3},({{\rm{r}}}^{2}=0.993)\end{array}$$

and

7 $$\begin{array}{ccc}equatorial\,length\,of\,the\,eyeball & = & -7.70+2.57\times day\,of\,incubation-0.1294\\  &  & \times \,day\,of\,incubatio{n}^{2}+0.002283\\  &  & \times \,day\,of\,incubatio{n}^{3},({{\rm{r}}}^{2}=0.997)\end{array}$$

with emphasized excellent fitting.

**Figure 7:**
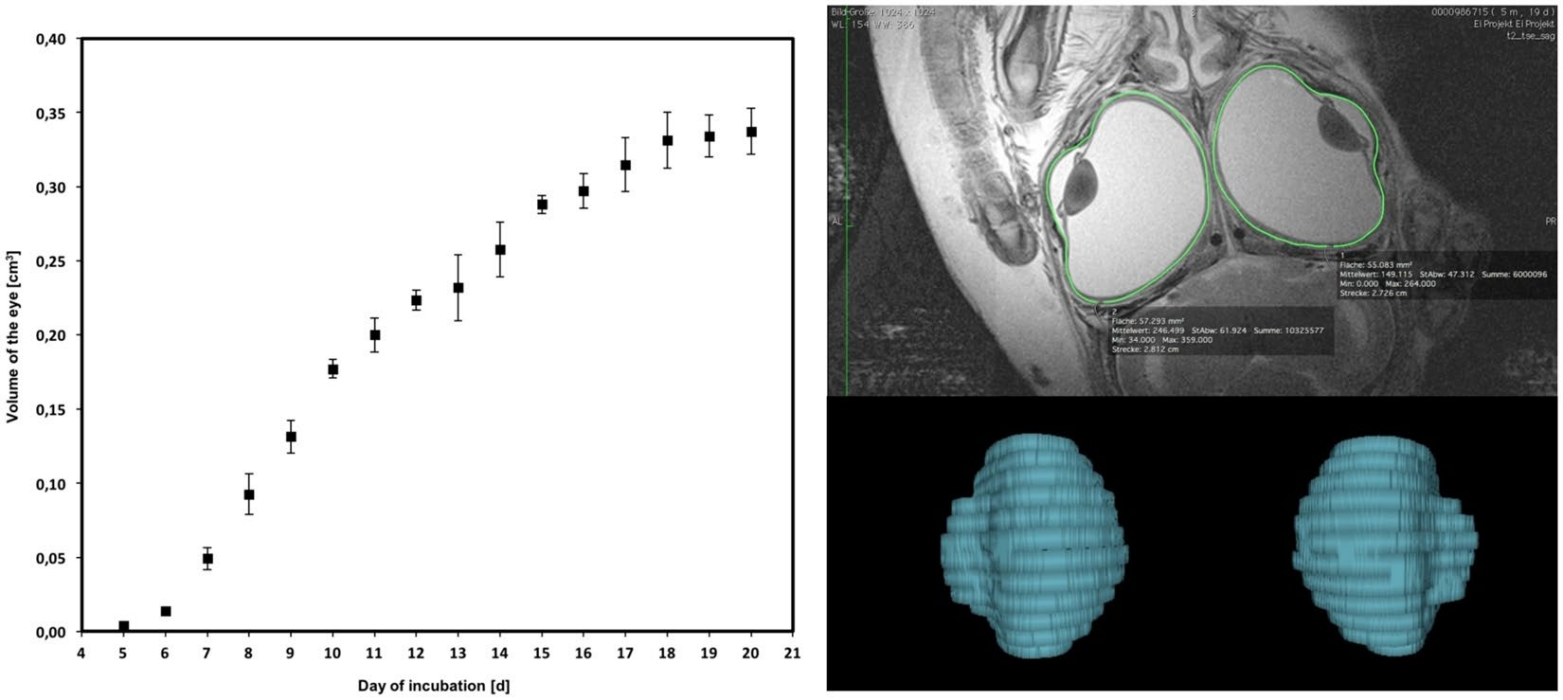
Volume of the eyeball from E5 to E20, as measured by MRI.

The volume of the eyeball (Fig. 7) was calculated using the OsiriX^©^ 3D-reconstruction functionality. Detectable eyeball volume increased more than 80-fold from 0.004 cm^3^ at E5 to 0.337 cm^3^ at E20 with almost linear volume growth from E5 to E10 and a lesser rate of growth during the second half of *in ovo* development from E10 to E20.

**Figure 8:**
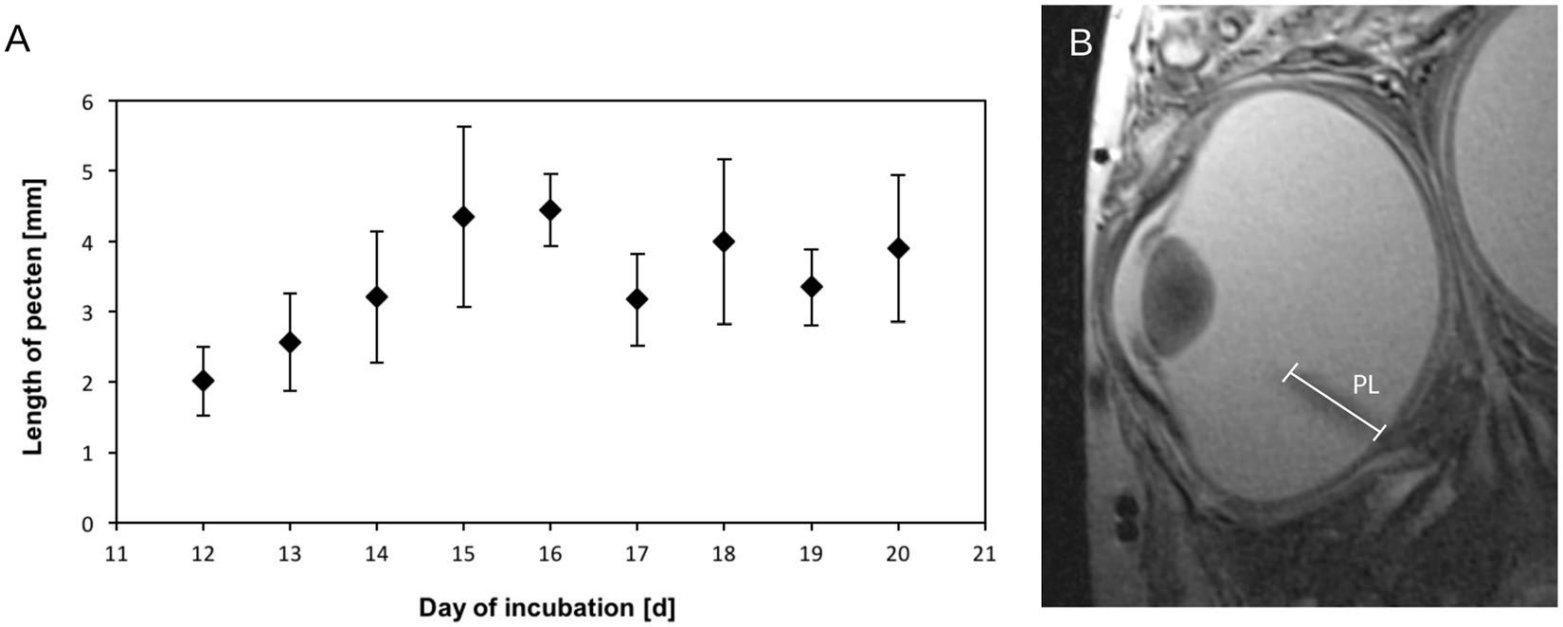
(**A**) Graph showing pecten length from E12 to E20. (**B**) Example to illustrate MRI measurement of pecten length.

The pecten oculi is an anatomic structure specific to the avian eye and contains numerous blood vessels for the nutritional support of the retina^18^. The pecten is a delicate structure arising from a primordium at the closing choroid fissure and projecting into the vitreous body, starting at E8. We were able to identify pecten structures at E10 and measured pecten length in our MR images from E12 onward, showing a moderate growth curve with relatively constant values after E15 (Fig. 8).

## Discussion

As frequently mentioned in the literature and confirmed again in the present study, MRI is an excellent tool for non-invasive and non-destructive imaging^1, 2, 8, 12, 13^, especially of the eye^15, 16^. MRI in human eyes correlates strongly with results of conventional ophthalmic imaging techniques^15, 16, 19^. To date, MRI has been used several times to study chick embryos^1, 2, 8, 12, 13^. To the best of our knowledge, those studies involved either *in vitro* imaging of chick embryos^8^ or merely covered parts of the embryonic period^1^ and in no case did they describe *in vivo* diagnostics from day 1 to day 20 of incubation^1, 2, 8, 13^. Our results suggest that a longitudinal study covering the entire embryonic period is feasible and that MRI is suitable for *in ovo* and *in vivo* investigations of chick embryos. Referring to *in ovo* diagnostics the eggshell does not present a barrier to MR imaging of the embryo inside^1, 2^.

Several approaches have been adopted for studying the appearance and development of chick organs or the vascular system using a variety MR modalities^1, 2, 13^. In our study, focusing on the eye, it was possible to image several intraocular structures from E5. Surface area, eyeball volume and lens thickness had been determined in a previous study^1^, but were reported to start at a later time point, namely at E12. In the present study, to obtain an accurate overview of chick embryonic eye growth, we measured five intraocular dimensions: axial and equatorial length (AL, EL), lens thickness and lens diameter (LT, LD), and length of vitreous body (VB) (Fig. 6), as well as the volume of the eyeball (Fig. 7) and the length of the pecten (PL) (Fig. 8). Almost linear growth was found for lens diameter and lens thickness during development from E5 to E20, while vitreous body length, and the axial and equatorial length of the eyeball showed higher growth rates until E10 followed by moderate growth thereafter.

The pecten oculi is a highly vascularized structure specific to the avian eye that has been implicated in perfusion and nutrition of the inner retina^20, 21^. The pecten primordium is of glial origin and extends into the vitreous body, forming many pleats during subsequent growth. In our study this unique structure was successfully monitored from E12 onward. At this developmental time point endothelial barrier properties are about to become established in the pecten and these are crucial for its later function^18^. While pecten growth and maturation continue beyond hatching, our results demonstrate that MRI permits monitoring of the growth and *in vivo* localization of this delicate supportive structure that is relevant to retinal nutrition.

In a further main enquiry addressed by this study, two groups (A and B) were compared to clarify whether daily MRI scanning, including cooling, has any effects on embryonic development. According to the study by Bain *et al*., the combination of repeated cooling and multiple MRI scans does not slow down or arrest the embryonic development of chicks^1^. However, that investigation started at E12 and did not consider cooling as well as MRI-based bioeffects in the early embryonic period. Our growth data for lens thickness and axial length for groups A and B indicate no significant difference between eggs scanned every day (Group A) and eggs scanned only at one single time point (Group B).

Several studies have investigated retinal neurogenesis, development or degeneration in various model systems by evaluation of cell numbers and distribution^22–24^. Ganglion cells are distributed unevenly across the mature retina and form specific patterns of high-density areas that differ among species, depending on habits and necessity^25^. Chickens usually possess only one of these high-density areas; however, it has been demonstrated that post-hatching chicks have two such areas^26^. Hence, the total number of cells varies significantly depending on the age of the chicks. Chen *et al*. reported an increase in total cell number in the chicken ganglion cell layer from 3.64 × 10^6^ at E8 to a maximal 7.85 × 10^6^ at E14. The number of cells decreased slightly to 6.08 × 10^6^ at post-hatching day 1 (P1) and 4.87 × 10^6^ at P8^27^. Because the retinae were stained with cresyl violet, the reported total cell numbers include retinal ganglion cells as well as a mixture of microglia and displaced amacrine cells. Galindo-Romero *et al*. recorded a relatively consistent number of 1.9 × 10^6^ retinal ganglion cells between E12 and P11 following Brn3a+ labeling^28^. Similarly, Rager & Rager estimated a number of about 2.4 × 10^6^ retinal ganglion cells, remaining constant from E18 until adulthood, following an electron microscope evaluation of optic nerve fibers^23^.

We estimated total cell counts (mean of 2 eyes) of 3.79 × 10^6^ (Group A) and 3.85 × 10^6^ (Group B) in the chick embryo retinal ganglion layer at E20 in both experimental conditions. We recognize that interneuron cells and glial cells will have been included alongside the projecting retinal ganglion cells. Nevertheless, from the estimated comparable cell numbers, we conclude that daily MRI, including cooling, seemingly does not interfere with neuronal, i.e., retinal development.

In terms of the MRI and histological findings in Groups A and B, all the chick embryos used in this study developed normally in accordance with reference criteria and it can be assumed that multiple MRI scans do not delay or modify development in the chick embryo.

## Conclusions

The present study describes a novel *in ovo* scan protocol for *in vivo* MR imaging of chick embryos over the entire prenatal period. Imaging covered the complete developmental period up to E20, and intraocular biometry was possible from E5 onward. Our data show that neither moderate cooling nor daily UHF-MRI examinations are harmful to the embryonic eye as revealed by MRI and histology. We conclude that MRI can be used to image the very smallest intraocular changes and has the capability to depict embryonic ocular development in a noninvasive and truly longitudinal manner.
